# Medical Help-Seeking Strategies for Perinatal Women With Obstetric and Mental Health Problems and Changes in Medical Decision Making Based on Online Health Information: Path Analysis

**DOI:** 10.2196/14095

**Published:** 2020-03-04

**Authors:** Kyungmi Chung, Hee Young Cho, Young Ran Kim, Kyungun Jhung, Hwa Seon Koo, Jin Young Park

**Affiliations:** 1 Institute of Behavioral Science in Medicine Yonsei University College of Medicine Seoul Republic of Korea; 2 Department of Psychiatry Yonsei University College of Medicine Severance Hospital, Yonsei University Health System Seoul Republic of Korea; 3 Department of Obstetrics and Gynecology CHA Bundang Medical Center CHA University School of Medicine Seongnam Republic of Korea; 4 Department of Psychiatry and Behavioral Neuroscience International St. Mary's Hospital Catholic Kwandong University Incheon Republic of Korea; 5 Fertility Center CHA Bundang Medical Center CHA University School of Medicine Seongnam Republic of Korea

**Keywords:** perinatal care, obstetrics, mental health, information seeking behavior, help-seeking behavior, self efficacy, health literacy, consultation, decision making, internet

## Abstract

**Background:**

Previous studies have revealed that most pregnant women rarely discuss informal information found on the internet with health professionals and have frequently expressed concerns for medical experts’ reactions to the online information they shared, as well as the lack of time to consult the medical experts in general. To date, little information is available on the effect of individual differences in utilizing medical help-seeking strategies on their medical decisions during the perinatal period.

**Objective:**

The objectives of this study were (1) to determine associations among perinatal women’s medical help-seeking strategies, changes in medical decision making, and online health information utilization with a focus on the mediating effect of self-efficacy in perinatal health literacy on the intent to consult health professionals, and (2) to clarify these associations in perinatal women with two different medical problems: obstetric and mental health.

**Methods:**

A total of 164 perinatal women aged 24 to 47 years (mean 34.64, SD 3.80) repeatedly completed the Problem Solving in Medicine and Online Health Information Utilization questionnaires to examine the moderating effect of two types of medical problems on their decision-making processes. To validate the hypothesized relationships in the proposed conceptual model encompassing obstetric and mental health problem-solving models, path analyses were performed.

**Results:**

This study found that some perinatal women, who use an online informal medical help-seeking (OIMH) strategy, would be more likely to change their medical decisions based only on internet-based information without consulting health professionals (*P*<.001), compared to other women using different medical help-seeking strategies. Particularly, this concern is significantly prevalent when encountering obstetric problems during the perinatal period (obstetric problem-solving: *P*<.001; mental health problem-solving: *P*=.02). Furthermore, perinatal women with mental health issues using the OIMH strategy showed a significant difference in intent to consult health professionals based on online health information when the medical problem they had to solve was different (obstetric problem-solving: *P*=.94; mental health problem-solving: *P*=.003).

**Conclusions:**

Despite the positive mediating effects of perinatal women’s enhanced health literacy on the intent to discuss personal medical issues with health professionals based on online health information, the strategy used is of fundamental importance for understanding their help-seeking and decision-making processes during the perinatal period. Beyond a short consultation to steer patients quickly and authoritatively towards an obstetric doctor’s choice of action, it is recommended in this study that obstetricians attempt to provide their patients with needed context for the information found online. To fully explain this information with an open mind, they should actively develop or support information and communications technology (ICT)-based health information services.

## Introduction

### Background

Medical help-seeking can be defined as the ability to actively seek help from others to cope with medical problems or painful experiences [1-3]. The help-seeking process is a transition from the personal domain (being aware of personal needs, thoughts, and feelings) to the interpersonal domain (being willing to share and disclose one’s needs to others) [2,3]. Internet health-seeking complements rather than replaces in-person health-seeking [4,5]. Particularly, online health information has been used to fill an informational void that promotes self-efficacy and affects medical decisions and actions about how to treat an illness or other medical conditions; this information can be discussed with friends, family members, and health care providers [6]. Physical and mental health professionals have serious concerns about the quality of online health information [6], and women in the perinatal period from conception through pregnancy to childbirth and the postpartum stage become more overwhelmed with false or misleading health information from multiple online sources, which in turn causes them to feel frightened and anxious rather than informed and empowered [7-11] and vise versa [12]. Even though internet searching allows pregnant women with the need for empirical medical information specific to their own situations to readily access experienced women’s birth stories on social media and provides social support to anxious women at the same time [13], women with a less experienced or robust capacity to make sense of conflicting health information from informal online sources may challenge their pre-existing ideations and aspirations of birth processes and address issues that they have not yet considered [11,14]. Hence, the provision of appropriate online health information can be considered an important step toward patient education and empowerment [6,15].

When it comes to dealing with difficulty assessing the accuracy, reliability, and credibility of relevant medical information on the internet, the evidenced-based information provided by health professionals will play a critical role in guiding perinatal women’s medical decisions on the right course of treatment [16]. For prenatal women, the association between trust in health providers and medical information sources should be carefully considered. Unlike many chronic disease patients who maintain the primacy of information acquisition from their doctors [17], mothers with lower levels of trust in health providers use more informal sources such as friends, family, other parents, the internet, and alternative medicine providers and do not regard their doctors as the main source of medical information [18]. Furthermore, most pregnant women have rarely discussed informal information found on the internet with health professionals [19], having frequently expressed concerns on medical experts’ negative reactions to the online information they brought to the outpatient clinic [20] as well as on the lack of time to consult them [21,22]. Without either perceiving self-efficacy in perinatal health literacy or contacting health professionals as the last bastion of medical decision based on online health information, women who solve medical problems by online informal consultation or search results can be more likely to make the wrong medical decisions, fail to receive appropriate medical treatments, or be confronted with new decision-making situations [23]. In this regard, this study focused on the association between perinatal women’s intentions of online informal medical help-seeking for health problems and their medical decision-making processes based on health information from the internet.

To address this issue, individual differences in using medical help-seeking strategies should be considered because the majority of pregnant women (83.7%) tend to use multiple information sources whose conflicting information increases anxiety and leads those with no chronic diseases to decide not to use medicines more than those with chronic diseases [24]. Based on the Chen and colleagues’ research model [25], this study categorizes perinatal women into four types of health information seekers with different medical help-seeking strategies, considering the formality (formal: querying doctors, medical experts, and health care professionals vs informal: querying family members, friends or relatives, and experienced people with previous medical problem-solving experiences) and channel (online: email, internet medical forum, instant messenger, and websites or online blogs vs nononline: face-to-face visit and communication) of the information source: (1) nononline formal medical help-seeking (NFMH), (2) online formal medical help-seeking (OFMH), (3) nononline informal medical help-seeking (NIMH), and (4) online informal medical help-seeking (OIMH). Further, when encountering two different obstetric and mental health problems, whether perinatal women with the four distinct medical help-seeking strategies will make medical decisions and change their decisions in a different manner, particularly based on online health information, has yet to be determined by comparing the proposed research model within the same Korean sample.

### Objectives

Given the potential impact of the information on a patient’s medical decision-making process that will lead to a change in their health-seeking behaviors, the personal, cognitive, and social skills that determine an individual’s motivation and ability to obtain, evaluate, and use health care information is of great importance for making appropriate medical decisions and promoting and maintaining good health [26]. The objective of this study was to develop and validate a medical problem-solving model to (1) determine associations among perinatal women’s medical help-seeking strategies, changes in medical decision making, and online health information use with a focus on the mediating effect of self-efficacy in perinatal health literacy on the intent to consult health professionals and (2) clarify these associations in perinatal women with two different medical problems (obstetric and mental health).

### Research Model and Hypotheses

**Figure 1:**
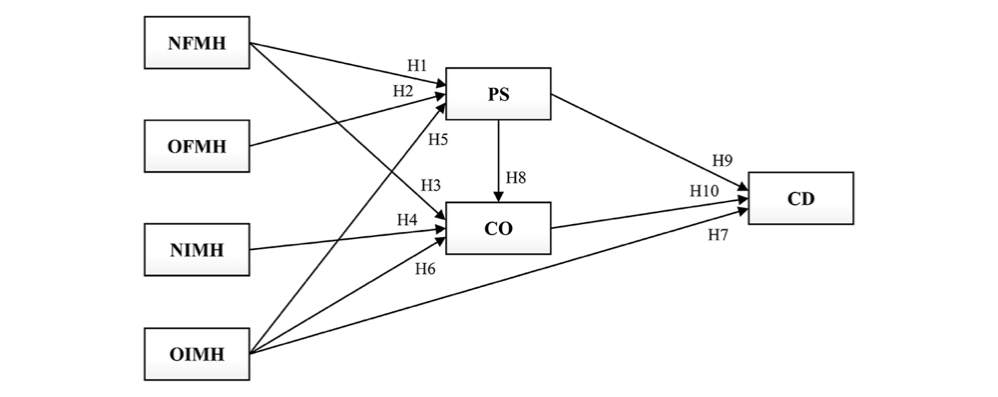
Medical problem-solving model. NFMH: nononline formal medical help-seeking; NIMH: nononline informal medical help-seeking; OFMH: online formal medical help-seeking; OIMH: online informal medical help-seeking; PS: promoting self-efficacy in perinatal health literacy based on online health information; CO: consulting others (health professionals) based on online health information; CD: changing their medical decisions based on online health information.

As shown in Figure 1, this study proposes a medical problem-solving model with a 2-step mediation approach that includes promoting self-efficacy in perinatal health literacy (PS) and consulting others (CO), both based on online health information perinatal women obtained, as potential mediators of the effects of perinatal women’s medical help-seeking strategies on changes in their medical decisions based on online health information (CD). Furthermore, the moderating role of two types of medical problems (obstetric vs mental health) will be further examined in this conceptual research model. According to the model, the following hypotheses are stated in the literature review.

In terms of the formality of medical help-seeking strategies, patients who prefer to seek medical help from formal sources have good health literacy [27]. Particularly, patients seeking online formal medical help achieve better health literacy [28] than those seeking nononline formal medical help in that the OFMH strategy is beneficial to compare, cross-check, and evaluate health-related information from multiple reliable information sources by looking for consistent results. However, as far as patients’ trust in their doctors is concerned, perinatal women with high trust levels will promote self-efficacy in perinatal health literacy based on their doctors’ guidelines, while those with low trust levels will do so based on online health information because they do not regard their doctors as the main source of medical information [18]. Thus, we posit the following hypotheses:

- H1/H2: Perinatal women’s strategies of (1) NFMH and (2) OFMH will be positively associated with PS.

Additionally, a recent study of patient samples, not specifically consisting of perinatal women, revealed that patients employing an NFMH strategy when facing medical problems had a significantly positive association with consulting others about relevant issues based on the online health information they obtained [25]. In the perinatal period, women who have maintained a long-term doctor-patient relationship through rapport building can be motivated to directly address their medical concerns sourced from the internet in order to reduce anxiety before and after a visit to the health professional [19,20,29-34]. As a facilitating factor in the decision to communicate with health professionals about difficulties in the perinatal period [35], a woman’s social network (eg, partner, friends, relatives, family members, and experienced mothers) will influence the help-seeking process by encouraging professional help-seeking, participating in the decision-making process, and initiating help-seeking [36]. Accordingly, the following hypotheses are proposed:

- H3/H4: Perinatal women’s strategies of (3) NFMH and (4) NIMH will be positively associated with CO.

Among the various benefits of using the internet, providing perinatal women with personal and emotional support from other women in similar situations is of unquestionable importance [13], since they are vulnerable to undergoing unknown, unpredictable events. In fact, finding people with the same medical problems is not easy online, but it will be helpful to share their concerns and doubts with other women and take positive energy from one another in online forums and social media platforms [37], thereby reducing anxiety and increasing confidence in making medical decisions for themselves and their babies after such internet use [20,21]. In addition, the internet can help expert users identify more questions they wanted to ask a health professional, be more involved in the decision-making process, make better decisions, and feel they had more control over their decisions than novice users [21]. Hence, we postulate the following hypotheses:

- H5: The perinatal women’s strategy of OIMH will be positively associated with PS.
- H6: The perinatal women’s strategy of OIMH will be positively associated with CO.
- H7: The perinatal women’s strategy of OIMH will be positively associated with CD.

According to a continuum of health literacy including basic or functional health literacy, communicative/interactive health literacy, and critical health literacy, there is a need to focus more on developing the skills and confidence to make well-informed health choices than on limiting the transmission of information [38]. The internet can empower people, including pregnant women, to communicate with their health professionals and play a critical role in making decisions [21]. However, the association between promoting self-efficacy in perinatal health literacy and changing perinatal women’s medical decisions might interfere with their anxiety levels. First-time pregnant women show a significant negative association between the level of maternal anxiety and feelings of control during labor [39]. As fears can be caused by negative moods and stories told by others about alarming information, diseases, and child-related problems in primiparas as well as negative experiences of a previous pregnancy, childbirth, and a baby’s health and care in multiparas, it will not be easy to change their views to align with the online health information they obtained without communicating with health professionals [40]. Therefore, we hypothesize the following:

- H8: PS will be positively associated with CO.
- H9: PS will be negatively associated with CD.
- H10: CO will be positively associated with CD.

## Methods

### Participant Recruitment

To test the proposed hypotheses, we administered an internet-based questionnaire built with Google Forms (Google Inc). Perinatal women aged 19 to 59 years who were being prepared for pregnancy, in fertility treatment, pregnant, or postpartum (up to 2 years after delivery) and had the ability to read, understand, and respond to the questionnaire items were eligible to be enrolled in this study. Those who failed to meet the inclusion criteria were not listed in our survey pool. In total, 168 women responded to the questionnaire for a 50.5% response rate, as the total survey pool consisted of 333 patients from the Fertility Center and/or Department of Obstetrics Gynecology, CHA Bundang Women’s Hospital, South Korea. This study was approved by the institutional review board of CHA Bundang Medical Center, CHA University.

The questionnaire was delivered to the participants’ mobile phones via a Google Forms link embedded in a short message service or multimedia messaging service text message or chat in KakaoTalk (Kakao Corp). Participants were allowed to access the survey link during a 2-month period from November 1, 2018, to December 31, 2018. To identify participants who met the criteria, a unique identification (ID) number was assigned to each patient with the survey link, and all patients were instructed to enter the preassigned ID number into the Google Form. Before receiving access to the online questionnaires, patients were asked to read and understand the aims of this study and relevant information, confirming that participating in the survey would be considered the equivalent of giving informed consent.

### Measurement Instruments

To assess the associations between perinatal women’s strategies of medical help-seeking to solve obstetric and mental health problems, medical decision making, and their online medical information use, the Problem Solving in Medicine and Online Health Information Utilization questionnaires developed by Chen and colleagues [25] were implemented twice in this study. The wording of the original questionnaire items was slightly modified to reflect the context of medical problems to be solved.

The Problem Solving in Medicine questionnaire consisted of 2 constructs with 6 observed variables measuring 27 latent variables: medical help-seeking (NFMH: 4 items, OFMH: 4 items, NIMH: 5 items, and OIMH: 5 items) and health information searching (nononline health information search [4 items] and online health information search [5 items]). The Online Health Information Utilization questionnaire included 2 constructs with 3 observed variables measuring 12 latent variables: medical decision making (PS: 4 items and CO: 4 items) and changes in medical decision making (CD: 4 items) based on online health information. All items were rated on a 7-point Likert scale ranging from 1=strongly disagree to 7=strongly agree. The reliability, validity (Table 1), and descriptive statistics (Table 2) were assessed for the measurement instruments.

Pregnancy-related anxiety was measured with a 10-item scale [41] that assessed the extent to which women felt concerned about their health, their baby’s health, labor and delivery, and caring for a baby. In this study, responses were made on a 5-point Likert scale ranging from 1=not at all to 5=very much. Items on the pregnancy-related anxiety scale were as follows: (1) I am confident of having a normal childbirth (reverse-coded), (2) I think my labor and delivery will go normally (reverse-coded), (3) I have a lot of fear regarding the health of my baby, (4) I am worried that the baby could be abnormal, (5) I am afraid that I will be harmed during delivery, (6) I am worried about how the baby is growing and developing inside me, (7) I am worried about losing the baby, (8) I am worried about having a hard or difficult labor and delivery, (9) I am worried about taking care of a new baby, and (10) I am worried about developing medical problems during my pregnancy.

**Table 1:** The internal reliability and convergent validity of the measurement instruments.

Factor and item		Factor loading	Cronbach alpha	Composite reliability^a^	Average variance extracted
**NFMH^b^**					
	NFMH1	0.84	.80	0.86	0.67
	NFMH2	0.79	—	—	—
	NFMH3	0.83	—	—	—
**OFMH^c^**					
	OFMH1	0.84	.85	0.85	0.58
	OFMH2	0.80	—	—	—
	OFMH3	0.76	—	—	—
	OFMH4	0.64	—	—	—
**NIMH^d^**					
	NIMH2	0.85	.83	0.87	0.68
	NIMH4	0.72	—	—	—
	NIMH5	0.90	—	—	—
**OIMH^e^**					
	OIMH1	0.71	.86	0.86	0.55
	OIMH2	0.66	—	—	—
	OIMH3	0.72	—	—	—
	OIMH4	0.82	—	—	—
	OIMH5	0.78	—	—	—
**PS^f^**					
	PSOHI1	0.68	.86	0.89	0.67
	PSOHI2	0.89	—	—	—
	PSOHI3	0.87	—	—	—
	PSOHI4	0.82	—	—	—
**CO^g^**					
	COOHI1	0.59	.78	0.83	0.62
	COOHI2	0.89	—	—	—
	COOHI4	0.85	—	—	—
**CD^h^**					
	CDOHI1	0.83	.93	0.94	0.80
	CDOHI2	0.91	—	—	—
	CDOHI3	0.93	—	—	—
	CDOHI4	0.91	—	—	—

^a^Composite reliability is defined for each factor by the ratio of the total variance (squared sum of standardized factor loadings for items) to the observed variance (squared sum of standardized factor loadings for items + sum of error variances). As Cronbach alpha is sensitive to the number of items in the scale and tends to underestimate the internal consistency reliability except under restricted assumptions of equal common factor loadings and uncorrelated measurement errors, composite reliability is measured as the second check for internal consistency.

^b^NFMH: nononline formal medical help-seeking.

^c^OFMH: online formal medical help-seeking.

^d^NIMH: nononline informal medical help-seeking.

^e^OIMH: online informal medical help-seeking.

^f^PS: promoting self-efficacy in health literacy based on online health information (PSOHI).

^g^CO: consulting others based on online health information (COOHI).

^h^CD: changing medical decisions based on online health information (CDOHI).

**Table 2:** Descriptive statistics and discriminant validity of the measurement instruments.

Factor	Mean (SD)	NFMH^a^	OFMH^b^	NIMH^c^	OIMH^d^	PS^e^	CO^f^	CD^g^
NFMH	5.44 (1.26)	0.82^h^	—	—	—	—	—	—
OFMH	3.44 (1.56)	0.30	0.76^h^	—	—	—	—	—
NIMH	4.82 (1.41)	0.25	0.13	0.83^h^	—	—	—	—
OIMH	2.95 (1.43)	0.09	0.57	0.30	0.74^h^	—	—	—
PS	4.29 (1.11)	0.19	0.30	0.18	0.27	0.82^h^	—	—
CO	4.84 (1.07)	0.25	0.19	0.25	0.24	0.33	0.79^h^	—
CD	4.09 (1.28)	0.05	0.08	0.15	0.26	0.03	0.28	0.90^h^

^a^NFMH: nononline formal medical help-seeking.

^b^OFMH: online formal medical help-seeking.

^c^NIMH: nononline informal medical help-seeking.

^d^OIMH: online informal medical help-seeking.

^e^PS: promoting self-efficacy in health literacy based on online health information.

^f^CO: consulting others based on online health information.

^g^CD: changing medical decisions based on online health information.

^h^Diagonal elements represents the square roots of the average variance extracted.

Kaisor-Meyer-Olkin and Bartlett sphericity tests, conducted to measure the sampling adequacy, showed that the samples met the criteria for factor analysis (0.83, *P*<.001). According to the results of an exploratory factor analysis using SPSS Statistics 18.0 software (IBM Corp) with varimax rotation on these 27 items, online health information search and nononline health information search were not clearly defined by the intended items and were excluded from the data analysis. We eliminated the following 4 items with loading values less than 0.50: NFMH4 (When I have an obstetric or mental health problem, I will ask the pharmacist of a nearby pharmacy store for information), NIMH1 (When I have an obstetric or mental health problem, I will seek help from a drugstore), NIMH3 (When I have an obstetric or mental health problem, I will go to the temple praying to god for advice), and COOHI3 (I will discuss relevant issues with my family or friends based on the health information on the internet). On the basis of the results of the exploratory factor analysis, the factor CO, which is an abbreviation for consulting others including a doctor (COOHI2), family members or friends (COOHI3), and other medical experts (COOHI4), did not include the item COOHI3, indicating that perinatal women of this study sample did not consider their family and friends as an important reference when making their medical decisions.

## Results

### Participant Characteristics

For the data analysis, 4 out of the 168 respondents were excluded (2.4%): 2 did not enter their given IDs and 2 did not have easy access to the internet when needed. A total of 164 perinatal women were included ranging in age from 24 to 47 years, with a mean age of 34.64 (SD 3.80) years. Perinatal women showed a mild to moderate level of fear and anxiety about their own health, their baby’s health, and labor and delivery outcomes (mean 2.85 [SD 0.69]), and the majority of them were aged from 30 to 49 years (150/164, 91.5%). As presented in Table 3, the sociodemographic and clinical characteristics of this study sample were documented as well as relevant information-seeking data more in detail.

**Table 3:** Sociodemographic and clinical characteristics of this study sample (n=164).

Characteristics		Participants, n (%)
**Gender**		
	Female	164 (100)
**Age of perinatal women in years**		
	19-29	14 (8.5)
	30-39	134 (81.7)
	40-49	16 (9.8)
**Age of their partners in years**		
	19-29	8 (4.9)
	30-39	116 (70.7)
	40-49	39 (23.8)
	50-59	1 (0.6)
**Marital status**		
	Unmarried	2 (1.2)
	Married	162 (98.8)
**Highest level of education completed**		
	High school	14 (8.5)
	Technical college (2-3 years)	37 (22.6)
	Undergraduate degree (bachelor’s: 4-5 years)	76 (46.3)
	Postgraduate degree (master’s)	32 (19.5)
	Postgraduate degree (PhD)	5 (3.0)
**Religious status**		
	Nonreligious	69 (42.1)
	Christian (Protestant)	49 (29.9)
	Christian (Catholic)	27 (16.5)
	Buddhist	19 (11.6)
**Professional status**		
	Unemployed (housewife)	72 (43.9)
	Employed	92 (56.1)
**Parity**		
	Nullipara	8 (4.9)
	Primipara	120 (73.2)
	Multipara	36 (22.0)
**Fetal plurality**		
	Before birth	23 (14.0)
	Singleton	131 (79.9)
	Twins	8 (4.9)
	Singleton + twins	2 (1.2)
**Number of children**		
	Before birth	23 (14.0)
	1	97 (59.1)
	2	39 (23.8)
	3	4 (2.4)
	4	1 (0.6)
**Number of sources for searching pregnancy/delivery information**		
	One	13 (7.9)
	Multiple	151 (92.1)
**Most important aspect of pregnancy/delivery information seeking**		
	Accuracy and reliability of information	115 (70.1)
	Diversity of information	13 (7.9)
	Recency of information	4 (2.4)
	Use of information	3 (1.8)
	Ease of use of media	25 (15.2)
	Interactivity of media	4 (2.4)
**Digital device used for information seeking (multiple response question)**		
	Computer (desktop/laptop)	34 (16.8)
	Tablet	7 (3.5)
	Smartphone	161 (79.7)

### Measurement Model

A confirmatory factor analysis performed with SPSS Amos 18.0 software (IBM Corp) indicated that the measurement model provided an acceptable fit to the data (χ^2^_278.00_=686.03, normed chi-square [χ^2^/df]=2.47, comparative fit index [CFI] 0.92, Tucker-Lewis index [TLI] 0.90, standardized root mean square residual [SRMR] 0.06, and root mean square error of approximation [RMSEA] 0.07). All values with an adequate level of construct validity were achieved by the recommended fit indices of the measurement model: χ^2^/df<3.0 [42], CFI>0.90 [43], TLI>0.90 [44], SRMR<0.08 [45], and RMSEA<0.08 [46].

The reliability and validity results (Tables 1 and 2) indicated that the questionnaire items had acceptable internal consistency with Cronbach alpha>.70 and composite reliability values>0.60, convergent validity values with factor loadings (all significant at *P*<.001) and average variance extracted values>0.50, and discriminant validity with the square roots of the average variance extracted values being higher than the correlations between the factors.

### Structural Model and Hypothesis Testing

**Figure 2:**
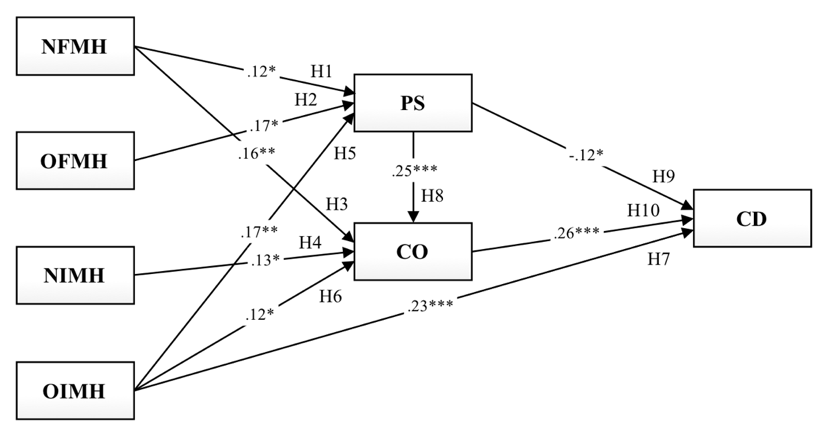
Medical problem-solving model with standardized path coefficients (*P<.05, **P<.01, ***P<.001). NFMH: nononline formal medical help-seeking; NIMH: nononline informal medical help-seeking; OFMH: online formal medical help-seeking; OIMH: online informal medical help-seeking; PS: promoting self-efficacy in perinatal health literacy based on online health information; CO: consulting others (health professionals) based on online health information; CD: changing their medical decisions based on online health information.

To validate the hypothesized relationships in the proposed conceptual model (Figure 1), path analyses were performed. As mentioned previously, the same-fit indices of the measurement model were used to assess the goodness of fit of structural models. The results of the path analysis for pregnant women’s medical help-seeking strategies, decision making, and any changes based on online information showed that the conceptual model had satisfactory levels of fit indices: χ^2^_5.00_=5.07, *P*=.41, χ^2^/df=1.01, CFI 1.00, TLI 1.00, SRMR 0.02, and RMSEA 0.01. As predicted in H1, H2, and H5, the strategies of NFMH (H1: *β*=.12, *P*=.02), OFMH (H2: *β*=.17, *P*=.01), and OIMH (H5: *β*=.17, *P*=.009) had positive effects on PS. Consistent with H3, H4, and H6, the strategies of NFMH (H3: *β*=.16, *P*=.003), NIMH (H4: *β*=.13, *P*=.02), and OIMH (H6: *β*=.12, *P*=.03) had positive effects on CO. Among those strategies for medical help-seeking, OIMH (H7: *β*=.23, *P*<.001) appeared as the only significant determinant of CD. H8, H9, and H10 were also supported by the results as CO was positively affected by PS (H8: *β*=.25, *P*<.001); however, CD was negatively affected by PS (H9: *β*=–.12, *P*=.03) and positively affected by CO (H10: *β*=.26, *P*<.001; Figure 2).

In addition, the other structural model for the moderating effect of different types of medical problems that pregnant women might encounter showed a satisfactory model fit: χ^2^_10.00_=10.34, *P*=.41, χ^2^/df=1.03, CFI 1.00, TLI 1.00, SRMR 0.02, and RMSEA 0.01. The results of the multiple group path analysis (Table 4) revealed that the only significant difference was found in 2 paths, H6_1 and H6_2, for obstetric and mental health problem-solving models, respectively (OIMHCO; critical ratio [CR] 2.01, significant at *P*<.05 [if CR>1.96]).

**Figure 3:**
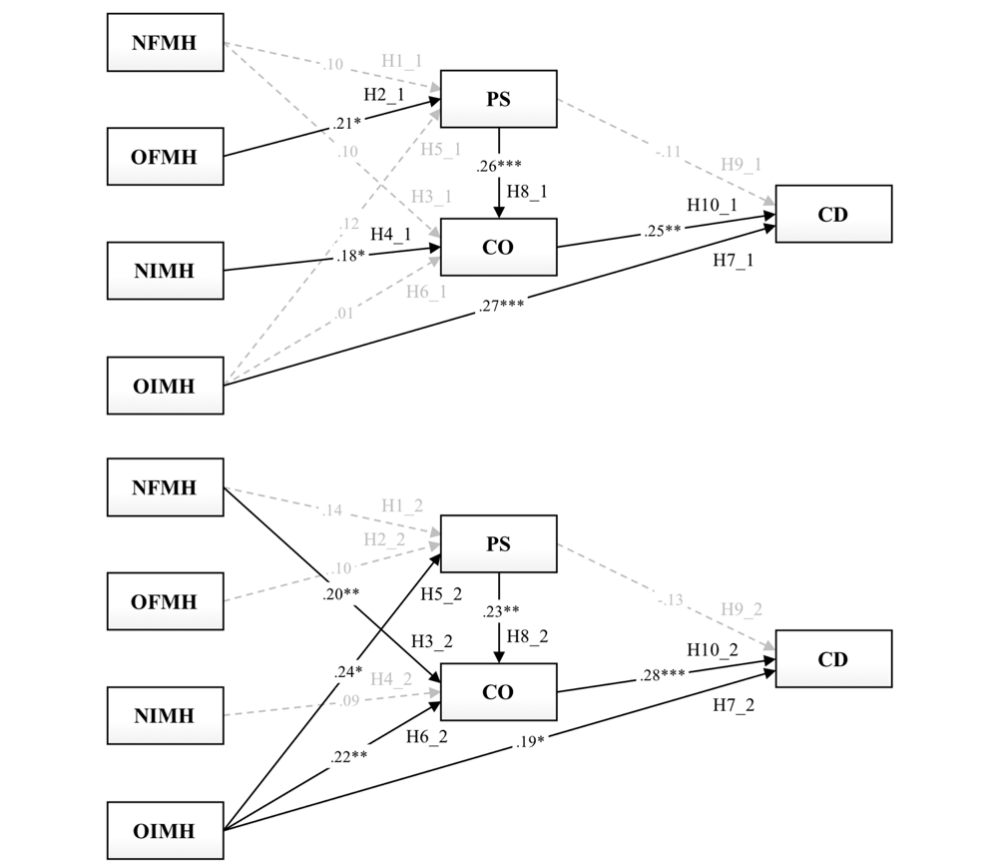
Obstetric (top) and mental health (bottom) problem-solving models with standardized path coefficients (*P<.05, **P<.01, ***P<.001). NFMH: nononline formal medical help-seeking; NIMH: nononline informal medical help-seeking; OFMH: online formal medical help-seeking; OIMH: online informal medical help-seeking; PS: promoting self-efficacy in perinatal health literacy based on online health information; CO: consulting others (health professionals) based on online health information; CD: changing their medical decisions based on online health information.

In the obstetric problem-solving model, the following hypotheses were rejected: H1_1 (*β*=.10, *P*=.20), H3_1 (*β*=.10, *P*=.18), H5_1 (*β*=.12, *P*=.17), H6_1 (*β*=.01, *P*=.94), and H9_1 (*β*=–.11, *P*=.15). As hypothesized, H2_1 (*β*=.21, *P*=.02) and H4_1 (*β*=.18, *P*=.02) were supported. In the mental health problem-solving model, the following hypotheses were rejected: H1_2 (*β*=.14, *P*=.07), H2_2 (*β*=.10, *P*=.29), H4_2 (*β*=.09, *P*=.24), and H9_2 (*β*=–.13, *P*=.10). Unlike the results of hypothesis testing for the obstetric problem-solving model, H3_2 (*β*=.20, *P*=.007), H5_2 (*β*=.24, *P*=.01), and H6_2 (*β*=.22, *P*=.003) were supported. In both models, the findings indicate that online health information was considered an important reference only for those with an OIMH strategy (H7_1: *β*=.27, *P*<.001; H7_2: *β*=.19, *P*=.02) when changing their medical decisions. Based on the online health information use, PS had positive effects on CO (H8_1: *β*=.26, *P*<.001; H8_2: *β*=.23, *P*=.002); furthermore, CO had positive effects on CD (H10_1: *β*=.25, *P*=.001; H10_2: *β*=.28, *P*<.001; Figure 3).

**Table 4:** Summary of the hypothesis testing results.

Model and hypotheses		*β* ^a^	*P* value	SE^b^	CR^c^	Supported
**Medical problem solving (n=328)**						
	H1: NFMH^d^→PS^h^	.12	.02	0.05	2.25	Yes
	H2: OFMH^e^→PS	.17	.01	0.05	2.56	Yes
	H3: NFMH→CO^i^	.16	.003	0.04	2.99	Yes
	H4: NIMH^f^→CO	.13	.02	0.04	2.36	Yes
	H5: OIMH^g^→PS	.17	.009	0.05	2.61	Yes
	H6: OIMH→CO	.12	.03	0.04	2.12	Yes
	H7: OIMH→CD^j^	.23	<.001	0.05	4.16	Yes
	H8: PS→CO	.25	<.001	0.05	4.72	Yes
	H9: PS→CD	–.12	.03	0.07	–2.16	Yes
	H10: CO→CD	.26	<.001	0.07	4.72	Yes
**Obstetric problem solving (n=164)**						
	H1_1: NFMH→PS	.10	.20	0.07	1.27	No
	H2_1: OFMH→PS	.21	.02	0.06	2.38	Yes
	H3_1: NFMH→CO	.10	.18	0.06	1.34	No
	H4_1: NIMH→CO	.18	.02	0.05	2.29	Yes
	H5_1: OIMH→PS	.12	.17	0.07	1.36	No
	H6_1: OIMH→CO^k^	.01	.94	0.06	0.07	No
	H7_1: OIMH→CD	.27	<.001	0.07	3.58	Yes
	H8_1: PS→CO	.26	<.001	0.07	3.42	Yes
	H9_1: PS→CD	–.11	.15	0.09	–1.43	No
	H10_1: CO→CD	.25	.001	0.10	3.24	Yes
**Mental health problem solving (n=164)**						
	H1_2: NFMH→PS	.14	.07	0.07	1.83	No
	H2_2: OFMH→PS	.10	.29	0.07	1.05	No
	H3_2: NFMH→CO	.20	.007	0.06	2.68	Yes
	H4_2: NIMH→CO	.09	.24	0.06	1.17	No
	H5_2: OIMH→PS	.24	.01	0.07	2.50	Yes
	H6_2: OIMH→CO^k^	.22	.003	0.06	2.96	Yes
	H7_2: OIMH→CD	.19	.02	0.07	2.38	Yes
	H8_2: PS→CO	.23	.002	0.08	3.12	Yes
	H9_2: PS→CD	–.13	.10	0.09	–1.64	No
	H10_2: CO→CD	.28	<.001	0.09	3.46	Yes

aβ: standardized coefficient.

^b^SE: standard error.

^c^CR: critical ratio.

^d^NFMH: nononline formal medical help-seeking.

^e^OFMH: online formal medical help-seeking.

^f^NIMH: nononline informal medical help-seeking.

^g^OIMH: online informal medical help-seeking.

^h^PS: promoting self-efficacy in health literacy based on online health information.

^i^CO: consulting others based on online health information.

^j^CD: changing medical decisions based on online health information.

^k^A multigroup path analysis was performed between H6_1 and H6_2.

Finally, mediation analyses were conducted by examining the direct and indirect effects of perinatal women’s medical help-seeking strategies and online health information use with CO and CD, with a focus on PS. It was revealed in the entire conceptual model that PS partially mediates the relation between CO and the strategies of NFMH (*β*=.03, *P*=.01) and OIMH (*β*=.04, *P*=.005), fully mediates the relation between OFMH and CO (*β*=.04, *P*=.003), and fully mediates the relation between NFMH and CD (*β*=.03, *P*=.049). In the obstetric problem-solving model, PS fully mediated the relation between the strategy of OFMH and CO (*β*=.06, *P*=.01), and in the mental health problem-solving model, PS partially mediated the relation between OIMH and CO (*β*=.06, *P*=.006).

## Discussion

### Principal Findings

As the goodness-of-fit indices between the measurement and structural models and the significance of all the hypothesized paths were statistically confirmed, the proposed conceptual model is expected to extend the understanding of the medical decision-making process in perinatal women with the 4 different medical help-seeking strategies. However, the decision-making process among perinatal women relied on which medical problem they would encounter, obstetrics or mental health, because there were substantial differences in the paths to self-efficacy in perinatal health literacy and intent to consult health professionals from their different medical help-seeking strategies, illustrated in the path diagrams (Figure 3). In the medical problem-solving model, the medical problem type had a partial moderating effect between OIMH and CO (H6_1 vs H6_2). Contrary to when the perinatal women using the OIMH strategy needed to solve mental health problems, no significant direct path from their adoption of the OIMH strategy to the intent to CO was found when they needed to solve obstetric problems. Furthermore, the direct paths from perinatal women’s adoption of the OIMH strategy to CD were significant in both obstetric and mental health problem-solving models.

When encountering obstetric problems, perinatal women with the OIMH strategy were more likely to change their medical decisions based on numerous conflicting and confusing online information sources without consulting health professionals compared with those with the same strategy to solve mental health problems. In this study, the majority of the perinatal women (151/164, 92.1%) preferred to search pregnancy/delivery information from multiple sources to compensate for a lack of accuracy and reliability of information, and only 4 out of the remaining 13 women (30.8%) preferred to obtain the information from doctors, nurses, and other health professionals, indicating they did not consider their doctors to be a main source of medical information, showed lower levels of trust in medical experts [18], and used multiple information sources whose conflicting information increases anxiety [24]. Considering that perinatal women were not willing to discuss the pregnancy-related information obtained from the internet with health professionals in the previous studies [16,19,31], they will be more exposed to the risks of either applying treatment that is not evidence-based or missing the right time to initiate the appropriate medical treatment with the best chance to achieve better pregnancy and delivery outcomes.

As evidenced by the findings of previous studies [21,35], perinatal women who use the NIMH strategy to solve obstetric problems tended to validate the online health information by communicating with health professionals. Lagan and colleagues [21] reported that almost all (96.2%) discussed the internet-based information with their partner or husband, and 70.8% discussed the information with at least one health professional, reflecting that the presence of a partner or relatives facilitates perinatal women’s communication with health professionals about their perceptions of being at risk [35]. Despite the associated support, informal help from partners, friends, and family members has disadvantages such as stigma or inappropriate support caused by their lack of relevant medical knowledge; therefore, making or changing medical decisions following suggestions provided by those with insufficient knowledge can be unhelpful or even harmful [47]. Meanwhile, those preferring to use the NFMH strategy did not make or change their medical decisions regarding obstetric problems by referring to online health information. As 70.1% of this study sample (115/164) considered accuracy and reliability to be the most important criteria in their evaluation when seeking pregnancy/delivery information, it could be inferred by our findings that the internet was considered a supplementary information source before or after contacting health professionals as a primary information source.

Our study revealed a positive relationship between self-efficacy in perinatal health literacy and intent to consult health professionals in both models. Once the level of perinatal women’s health literacy increases, they can be more easily encouraged to identify questions they wish to ask a health professional, be involved in the decision-making process, make better decisions, and exert more control over their decisions [21]. Searching online health information before paying a visit to their health professionals and after their consultation [30] is common as a means to deal with doubts and navigate other women’s pregnancy-related decisions, which can also result in increased confusion, anxiety, and fright [48]. With the perinatal women’s concerns on the lack of time to sufficiently discuss personal medical issues with health professionals [21,22] and the professionals’ reactions to the information they shared [20], a number of previous studies found that the majority of women believed that health professionals should suggest suitable and reliable internet websites [20,21] where they could find relevant information with appropriate context and avoid erroneous or misleading claims [48]. As revealed in the obstetric problem-solving model, the OFMH strategy was positively related to PS, which reflects the implications of other studies.

When it comes to solving mental health problems, perinatal women showed a different attitude toward online information use. Those using the NFMH strategy disclosed their intent to discuss relevant issues with mental health professionals without the precondition of enhanced self-efficacy in perinatal health literacy. Generally, psychiatrists and other mental health professionals, whose goals are to provide each patient with the opportunity to discuss problems with a sympathetic listener by helping to establish a rapport between the patient and doctor, educate and motivate the patient, and take a clinical examination of the mental state, depend on a psychiatric interview. In comparison with the significance of the direct and indirect paths from NFMH to CO (ie, NFMHPS, NFMHPSCO, and NFMHCO) in the two different medical problem-solving models shown in Figure 3 and Table 4, the findings of this study suggest that what women expected from the interaction with obstetricians or psychiatrists during their clinic visits would be different during the perinatal period. Accordingly, these findings can well explain why it is important for health professionals not to discourage their patients to share obstetric concerns and treatment suggestions retrieved from the internet.

### Limitations and Future Direction

There are several limitations of this study that should be considered in future research. The sample size and its demographic composition limited our opportunity to examine the possible age difference, relationship with one’s doctor, number of births, and history of experienced obstetric and mental health problems because this study focused on the entire perinatal period, encompassing conception, pregnancy, childbirth, and postpartum. Given the small sample size, it would result in an insufficient statistical power, particularly as reflected by significantly low standardized path coefficients. For a more accurate estimate, a larger sample size would be required in the future studies. In terms of age distribution, the proportion of participants aged 19 to 29 years (14/164, 8.5%) was considerably less than that of those aged 30 to 39 years (134/164, 81.7%). Given the age differences of people obtaining online health information [49], the possibility that younger women may differ in how they make and change their medical decisions by using online health information compared with older women cannot be ruled out. As this study sample, enrolled from a large hospital-based sample of patients, was willing to complete an online questionnaire, the participants might be more likely to have a good rapport with their doctors or be internet-savvy patients accessing the internet more frequently for solving medical problems than the others who did not so. Finally, whether perinatal women have given birth to at least one child or had a history of obstetric and mental health problems may be associated with their intent to either share their own experiences with other mothers in trouble or trust and follow the doctor’s instructions only. Taken together, there is a need to control for these determinants in future studies.

### Conclusions

Despite the positive mediating effects of perinatal women’s enhanced health literacy on the intent to discuss personal medical problems with health professionals, the findings from this study revealed that some women using an OIMH strategy would be more likely to change their medical decisions not by consulting health care professionals but by referring to online health information compared with other women using different medical help-seeking strategies. Particularly, this concern would be prevalent when encountering participants with obstetric problems during the perinatal period. Beyond a short consultation to quickly and authoritatively steer patients toward an obstetric doctor’s choice of action, it is recommended that obstetricians provide their patients with needed context for information found online. To fully explain this with an open mind, they need to actively develop or support information and communications technology–based health information services such as mobile apps and chatbots.

## Abbreviations

CD: changing their medical decisions based on online health information
CFI: comparative fit index
CO: consulting others (health professionals) based on online health information
CR: critical ratio
KHIDI: Korea Health Industry Development Institute
NFMH: nononline formal medical help-seeking
NIMH: nononline informal medical help-seeking
OFMH: online formal medical help-seeking
OIMH: online informal medical help-seeking
PS: promoting self-efficacy in perinatal health literacy based on online health information
RMSEA: root mean square error of approximation
SRMR: standardized root mean square residual
TLI: Tucker-Lewis index
